# *Mycobacterium tuberculosis* Drives Expansion of Low-Density Neutrophils Equipped With Regulatory Activities

**DOI:** 10.3389/fimmu.2019.02761

**Published:** 2019-11-27

**Authors:** Marco Pio La Manna, Valentina Orlando, Elvezia Maria Paraboschi, Bartolo Tamburini, Paola Di Carlo, Antonio Cascio, Rosanna Asselta, Francesco Dieli, Nadia Caccamo

**Affiliations:** ^1^Central Laboratory of Advanced Diagnosis and Biomedical Research, University of Palermo, Palermo, Italy; ^2^Department of Biomedicine, Neurosciences and Advanced Diagnostic, University of Palermo, Palermo, Italy; ^3^Department of Biomedical Sciences, Humanitas University, Milan, Italy; ^4^Department of Sciences for Health Promotion and Mother-Child Care “G. D'Alessandro”, University of Palermo, Palermo, Italy; ^5^Humanitas Clinical and Research Center–IRCCS, Milan, Italy

**Keywords:** tuberculosis, neutrophils, low density neutrophils, phagocytosis, transcriptomic analysis

## Abstract

In human tuberculosis (TB) neutrophils represent the most commonly infected phagocyte but their role in protection and pathology is highly contradictory. Moreover, a subset of low-density neutrophils (LDNs) has been identified in TB, but their functions remain unclear. Here, we have analyzed total neutrophils and their low-density and normal-density (NDNs) subsets in patients with active TB disease, in terms of frequency, phenotype, functional features, and gene expression signature. Full-blood counts from Healthy Donors (H.D.), Latent TB infected, active TB, and cured TB patients were performed. Frequency, phenotype, burst activity, and suppressor T cell activity of the two different subsets were assessed by flow cytometry while NETosis and phagocytosis were evaluated by confocal microscopy. Expression analysis was performed by using the semi-quantitative RT-PCR array technology. Elevated numbers of total neutrophils and a high neutrophil/lymphocyte ratio distinguished patients with active TB from all the other groups. PBMCs of patients with active TB disease contained elevated percentages of LDNs compared with those of H.D., with an increased expression of CD66b, CD33, CD15, and CD16 compared to NDNs. Transcriptomic analysis of LDNs and NDNs purified from the peripheral blood of TB patients identified 12 genes differentially expressed: *CCL5, CCR5, CD4, IL10, LYZ*, and *STAT4* were upregulated, while *CXCL8, IFNAR1, NFKB1A, STAT1, TICAM1*, and *TNF* were downregulated in LDNs, as compared to NDNs. Differently than NDNs, LDNs failed to phagocyte live *Mycobacterium tuberculosis* (*M. tuberculosis)* bacilli, to make oxidative burst and NETosis, but caused significant suppression of antigen-specific and polyclonal T cell proliferation which was partially mediated by IL-10. These insights add a little dowel of knowledge in understanding the pathogenesis of human TB.

## Introduction

Neutrophils are the most abundant cellular type of white blood cells in the body, and they should be considered to play a role in the first line of defense against infection. These cells are phagocytes that strongly react to inflammatory stimuli resulting in a massive infiltration to the site of infection through the chemokine CXCL8, which binds the receptors CXCR-1 and−2 (1, 2). In human tuberculosis (TB) neutrophils represent the most commonly infected phagocytes (3), however their role in TB protection and pathology still remains contradictory, with some data implicating neutrophils in the TB control and others associating them with TB pathology and disease progression (4, 5). *M. tuberculosis* bacilli are phagocyted by neutrophils and this feature has been demonstrated both by *in vitro* and *in vivo* studies (6–8). *In vivo* mice studies have demonstrated the presence of neutrophils 1-day post challenge with Bacille Calmette-Guerin (BCG) or *M. tuberculosis* in the lung tissue and in the airspaces of mice, where 1.6% of neutrophils contained mycobacteria (8). It was demonstrated in human lung tissues that 7% of the cells infected *in vitro* with various mycobacterial strains consisted of neutrophils (7). Their role has been significantly correlated with the control of *M. tuberculosis* infection in human blood (9), even if at the time of TB diagnosis the neutrophilia is associated with delayed clearance of bacteria from sputum (3). Indeed, the most frequent cells found in sputum and bronchoalveolar lavage from patients with active pulmonary TB are neutrophils.

Human studies further revealed that higher neutrophil counts are protective against early TB infection (9), thus, during the initial stages of infection, neutrophils play a protective role; however, a pathogenic role during the late stages of TB has been proposed (10). Neutrophilia has been assigned as a predictor of disease progression, pulmonary destruction, and even death (3, 5, 9, 11–14).

In some inflammatory disorders, a subset of low-density neutrophils (LDNs) that co-purify with peripheral blood mononuclear cells (PBMCs) in density gradient centrifugation has been identified (15), even if the origin and role of this subpopulation remain somewhat unknown. Some works have reported that LDNs display diverse profiles, they have been detected also in many other pathologies like sepsis, HIV infection, malaria, and, importantly, also in TB (16–19). Increasing the understanding of their surface marker patterns, cytokine expression, transcription factor regulators, and other trademarks of activation is of prime importance. Until now, despite several studies have described the diversity of neutrophil subpopulations, their functional role in the different pathologies remain still not fully elucidated.

Although LDNs have been already detected in TB patients, their frequency and clinical significance remain unclear. Therefore, in this paper, we have studied neutrophil cell compartment in patients with active TB disease, analyzing total neutrophils and their subsets in terms of frequencies, phenotype, functional features, and gene expression signature.

## Materials and Methods

### Characteristics of the Enrolled Individuals

A total of 149 individuals were prospectively enrolled as here reported and as described in detail previously (20): (a) Healthy Donors (H.D.): 40 individuals tested TST and QFT-IT-negative (11 men, 29 women; median age 44 years); (b) active TB disease: 71 individuals diagnosed with active pulmonary TB (with a positive *M. tuberculosis* culture from sputa or bronchoalveolar lavage; 54 men, 17 women, median range 31 years) who started specific treatment <8 days before enrolment (Table 1); (c) 38 cured TB patients (with a previous microbiological diagnosis; 17 women and 21 men, median age 42). Patients were recruited from the Department of Infectious Disease, University Hospital of Palermo. Active TB diagnosis was confirmed by bacteriological isolation of *M. tuberculosis* in 71 patients. All patients were treated in accordance with Italian guidelines and received therapy for 6 months. Treatment was successful in all participants, all of whom completed the full course of anti-TB chemotherapy. None of the TB patients had evidence of HIV infection or was being treated with steroid or other immunosuppressive or anti-tubercular drugs at the time of their first sampling. The study was approved by the Ethical Committee of the University Hospital in Palermo (approval number 13/2013), where the patients were recruited. Informed consent was signed by all participants. QFT-IT was performed as for manufactures instructions.

**Table 1:** Characteristics of study groups.

	**Healthy donors**		**Active TB**		**Cured TB**		**Total**	
Enrolled subjects (%)	40	(26.8%)	71	(47.7%)	38	(25.5%)	149	(100%)
Median age	44		31		42		40	
Range	23–62		17–73		17–70		17–82	
Male gender (%)	11	(27.5%)	54	(77.5%)	21	(55.3%)	86	(57.7%)
Origin (%)								
Western Europe	40	(100%)	14	(19.7%)	12	(31.6%)	56	(37.5%)
Eastern Europe	0	(0.00%)	29	(40.8%)	9	(23.7%)	38	(25.5%)
Asia	0	(0.00%)	8	(11.3%)	3	(7.9%)	11	(7.3%)
Africa	0	(0.00%)	18	(25.3%)	11	(28.9%)	29	(19.4%)
South America	0	(0.0%)	2	(2.8%)	3	(7.9%)	5	(3.3%)
TST (%)								
Positive	0	(0.00%)	15	(21.2%)	0	(0.00%)	15	(10.0%)
Negative	0	(0.00%)	4	(5.6%)	0	(0.00%)	4	(2.7%)
N.D.	40	(100%)	52	(73.3%)	38	(100%)	130	(87.2%)
IGRA TEST (%)								
Positive	0	(0.0%)	62	(87.4%)	33	(86.8%)	95	(63.7%)
Negative	14	(35.0%)	5	(7.0%)	2	(5.3%)	21	(14.1%)
Indeterminate	0	(0.0%)	2	(2.8%)	1	(2.6%)	3	(2.0%)
N.D.	26	(65.0%)	2	(2.8%)	2	(5.3%)	30	(20.1%)
TB diagnosis (%)								
Microbiological diagnosis			71	100%			71	(47.7%)

### Full Differential Blood Counts

Full blood counts (FBC) of peripheral blood collected in ethylene-diamine tetra-acetic acid (EDTA) containing tubes were performed by one clinical diagnostic laboratory using a five-part differential hematology analyzer (Coulter 4.500, Germany). FBC measurement was under strict quality procedures including twice-daily high and low internal quality control, fortnightly quality controls done by the clinical laboratory QC scheme and annual quality assurance as part of clinical laboratory QC scheme. The laboratory is accredited by the Italian National Accreditation System in accordance with international standards ISO17025/2005 and ISO 15189/2007.

### LDNs Isolation From PBMCs

Samples of peripheral blood (5 mL) were drawn by venipuncture and collected into EDTA tubes. The PBMCs and LDNs fractions were isolated from 5 patients with active TB disease by density-gradient centrifugation.

The venous blood was diluted 1:2 with PBS and centrifuged on ficoll (Lympholyte, human cell separation media; Cederlane, Canada) in a 15-mL polystyrene conical centrifuge tube for 20 min at 770×g (at room temperature, RT).

PBMCs were carefully collected by aspiration from the plasma-ficoll interface and washed two times (500×g for 5 min) in RPMI 1640 medium (Euroclone, Pero, Italy) supplemented with 20 mM HEPES, 100 U/mL penicillin, 100 μg/mL streptomycin, then resuspended in 1 ml of complete RPMI medium (10% heat-inactivated FCS, 2 mM L-glutamine) in a 15-mL polystyrene conical centrifuge tube.

LDNs were sorted from PBMCs using Miltenyi immunomagnetic microbeads (Miltenyi Biotec, Auburn, CA, USA).

PBMCs were incubated with 20 μl/1 × 10^6^ cells of anti-CD15 moAb conjugated with R-PE (clone HI98; Becton Dickinson, USA) for 20 min at RT in the dark.

Stained PBMCs were then incubated with anti-PE microbeads (MiltenyiBiotec) and CD15^+^ cells were obtained by positive immunomagnetic separation using MS columns (MiltenyiBiotec), according to manufacturer's protocol.

### NDNs Isolation

After PBMC-gradient separation, NDNs were collected by aspiration from the ficoll-red blood cell pellet interface, cells were washed one time with 2 ml of PBS in a 15-mL polystyrene conical centrifuge tube (500×g for 5 min).

After washing, cells were resuspended in 100 μl of PBS and treated with 0.5 molar ammonium chloride (2 ml for 20 min) to obtain the lysis of the remaining red blood cells.

Cells were pelleted down by centrifugation (500×g for 5 min). After the lysis of erythrocytes, NDNs were washed two times with PBS (500×g for 5 min) and resuspended in 1 ml of complete RPMI medium.

All experiments were performed using fresh cells, immediately after processing. Trypan Blue Staining Cell Viability (Euroclone, Pero, MI) was used to determine the cell viability in this study. Cytospin preparations were prepared and treated with Wright-Giemsa stain for morphological neutrophils observation (Supplemental Figure 1).

### Flow Cytometry

PBMCs or granulocytes (10^6^ cells each) of 22 active TB patients were transferred to 12 × 75 mm tubes, washed once, and incubated with FcR blocking reagent (Miltenyi Biotec, Auburn, CA, USA) for 5 min.

Cells were then incubated for 30 min at RT with or without the following mixture of fluorescent-labeled anti-human monoclonal antibodies: CD66b FITC (Clone G10F5), CD15-PE (Clone HI98), HLA-DR-PerCP (Clone G46-6), CD13 PE-Cy7 (Clone WM159), CD33-APC (Clone WM53), CD11b-FITC (Clone M1/70), CD16 PE-Cy7 (Clone 3G8), and CD45 APC-H7 (Clone 2D1) (eBioscience, San Diego, CA, USA).

Cells were acquired on FACSCantoII (Becton Dickinson, USA) using the following gating strategy; initial gate was on granulocytes on the basis of forward-side scatters followed by selection of CD45 vs. SSC parameter, doublets exclusion and gate on CD33^+^dim and CD66b^+^ cells, and used for further analysis.

### T Cell Proliferation Assay

PBMCs isolated from TB patients as above described were stained with CFSE (Biolegend, San Diego, CA) at final concentration of 1 μM and subsequently stimulated with BCG at MOI of 10:1 in the presence of NDNs or LDNs (ratio neutrophils: PBMCs = 10:1) in complete RPMI 1640 medium. After 6 days of incubation, PBMCs were harvested and stained using monoclonal antibodies anti-CD3 PE-Cy7 (clone SK7), anti-CD8 APC (cloneSK1), and anti-CD4 APC-H7 (clone RPA-T4) all from BD (Becton Dickinson, USA) for 20 min at RT. After the incubation, cells were washed and resuspended in 500 μl of FACSFLOW and acquired to FACSCANTO II and analyzed by FlowJo software (version 6.1.1; Tree Star, Ashland, OR, USA).

To analyze proliferation, cells were gated on lymphocytes, followed by gating on CD3^+^ cells.

The proliferation was calculated as fold of change by dividing the percentage of proliferation of CD3^+^ T cells stimulated with BCG co-cultured with LDNs and NDNs to the percentage of proliferation of CD3^+^ T cells stimulated with BCG.

### ELISA Assay

IFN-γ assay was performed using an ELISA kit Invitrogen by Thermo Fisher Scientific (UK). A total of 5 × 10^4^ PBMCs were put in 96 round bottom wells and were stimulated overnight in the presence of BCG and in the presence of NDNs or LDNs at the final volume of 200 μl. In some experiments anti-IL-10 neutralizing moAb was added to the co-culture. After 24 h, the supernatants were collected and used for the ELISA assay. The value of IFN-γ concentration, expressed as pg/ml, obtained in the unstimulated cells was subtracted to the values of the cytokine found in the different experimental conditions. The detection limit of IFN-γ was of 2 pg/ml as indicated by the manufacturers.

### Bacterial Strains, Infection, and Evaluation of Phagocytosis and NETosis

*M. bovis* BCG Danish strain and *M. tuberculosis*-GFP were grown to early mid-log phase in Middlebrook 7H9 broth, supplemented with 10% albumin-dextrose-catalase and 0.5% glycerol at 37°C. The bacterial aggregates were shattered by gentle agitation with 3-mm-diameter glass beads. The resultant bacteria were diluted in PBS. The solution was left standing for 15 min before the supernatant was collected and adjusted to an OD600 of 0.5 (~10^7^ individual bacteria/mL).

For *in vitro* infection, purified NDNs and LDNs were infected by mycobacteria at the multiplicity of infection (MOI) of 10 (bacteria to neutrophils) for 3 h. After the incubation, non-ingested bacilli were removed by washing four times with PBS. The absence of the extracellular bacteria was assessed by auramine-rhodamine staining. Then, cells were spotted into a slide and stained with the monoclonal antibody anti-MPO-FITC (clone 5B8, BD) and Hoechst 33342 for nuclei staining (ThermoFisher Scientific). After the incubation, the cells were washed twice and NETosis was evaluated by confocal microscopy.

To assess the phagocytic activity of the two cellular neutrophil populations, after *in vitro* infection with *M. tuberculosis*-GFP, the cells were washed four times and stained with DAPI to identify the nuclei. All experiments involving bacterial handling was performed in class III biological safety laboratory.

### Oxidative Burst Assay

The oxidative burst of the neutrophils was measured using Dihydrorhodamine-123 (DHR-123; BD) staining, according to the manufacturer's protocol. Briefly, 100 μl of NDNs and LDNs neutrophil suspension cells (10^6^ cells/mL in RPMI+ 10% FCS) were initially put in 12 × 75 mm tubes on ice for 10 min and then stimulated for 30 min with Phorbol-Myristate Acetate (PMA, from Sigma Aldrich), at the final concentration 1.35 μM in pre-warmed bath 37°C. Unstimulated controls were incubated in the same conditions. After the incubation, DHR123 was added to each tube and the cells were incubated for further 10 min in the same conditions. Oxidative burst was evaluated by flow cytometry measuring the mean fluorescence intensity of oxidized rhodamine 123, of neutrophils gated on the base of FSC vs. SSC parameters.

### RNA Extraction

Pellet of human isolated NDNs and LDNs were extracted by the Maxwell^®^ RSC miRNA Tissue kit (Promega, Madison, USA), following the manufacturer's instructions. RNA concentration and quality were measured using the NanoDrop 2000 Spectrophotometer (Thermo Fisher Scientific).

### PCR Arrays

Total RNA (600 ng) was reverse transcribed using the RT^2^ First Strand Kit (SABiosciences, Qiagen, UK, https://www.qiagen.com/us/products/). A total of 84 human cytokines and chemokines were tested using the RT^2^ Profiler PCR Array Human Innate & Adaptive Immune Responses array (Qiagen) in LDNs and NDNs isolated from 8 TB patients. Semi-quantitative RT-PCRs were performed using a real-time PCR detection system (QuantStudio 7 Flex Real-Time PCR System; Thermo Fisher Scientific) with a two-step thermal cycling: 95°C for 10 min, followed by 40 cycles (95°C for 15 s, 60°C for 1 min). Data were analyzed using the RT^2^ Profiler PCR Array Qiagen data analysis software, and *ACTB* and *B2M*as housekeeping genes.

### Statistics

To calculate the neutrophil/lymphocyte (N/L) ratio, the absolute neutrophil count was divided by the absolute lymphocyte count. The median or geometric mean was used for descriptive statistics for each parameter. The non-parametric Kruskal-Wallis was performed comparing the medians of N/L ratio, significance was settled at *p* < 0.05. The relationship between variables was evaluated using the Spearman rank correlation test. A two-side *p* < 0.05 was considered statistically significant. Data were analyzed using the Statistic software (Statsoft, Ohio, USA) and GraphPad prism, version 5.0 (GraphPad Software, San Diego, CA, USA). Negative binomial regression model was used to estimate the association between active TB and explanatory variable. To evaluate the statistical significance differences of the expression of surface molecule markers on neutrophil population, the burst activity and the T cell proliferation, assessed by flow cytometry, a Mann-Whitney test was used and significance was settled for *p* < 0.05. Unless indicated otherwise, results are expressed as mean ± SEM.

## Results

### A High N/L Ratio Is Associated With Active TB Disease

**Figure 1:**
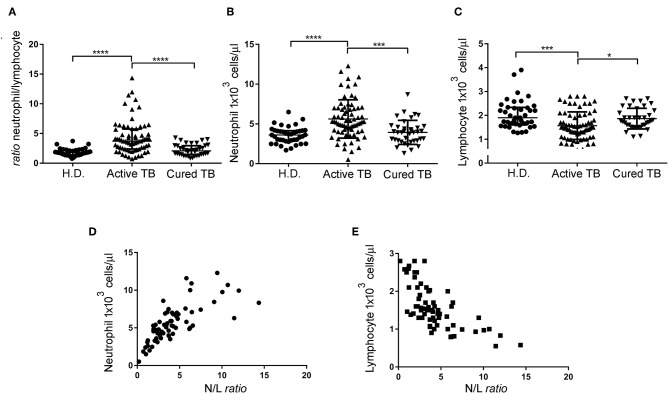
N/L ratio, neutrophil, and lymphocyte absolute count of the different cohort groups and correlation between the N/L ratio and absolute neutrophil and lymphocyte count. N/L ratio **(A)**, neutrophil absolute count **(B)**, and lymphocyte absolute count **(C)** of patients with active TB disease, cured TB patients, and H.D. Each dot represents the value of an individual subject. Each horizontal bar represents the median of each group. **(D)** Correlation between the N/L ratio and absolute neutrophil count, analyzed by Spearman rank correlation test. Data shown are the values of each individual subject. **(E)** Correlation between the N/L ratio and absolute lymphocyte count analyzed by Spearman rank correlation test. Data shown are the values of each individual subject. Significance of differences between groups was compared using Kruskal-Wallis test, ^*^p < 0.05, ^***^p < 0.001, ^****^p < 0.0001.

Previous studies have shown that the N/L ratio is a useful index in patients with active TB (11). Initially, we assessed in our study if N/L ratio is modulated by the clinical TB condition. As shown in Table 2 and Figure 1A, patients with active TB disease had a significantly (*p* < 0.0001) higher NL ratio (Median: 3.71, IQR: 2.38–5.74), compared to Healthy Donors (H.D.) (Median: 1.77, IQR: 1.37–2.16), which normalized after anti-mycobacterial therapy (Median: 2.08, IQR: 1.47–2.94). Conversely, no significant differences were observed among H.D., and cured TB patients.

**Table 2:** N/L ratio, neutrophil, and lymphocyte absolute count of the subjects enrolled.

**TB status**	**No of subjects enrolled**	**N/L** ***ratio***		**Neutrophil absolute count 1** **×** **10**^****3****^**/μl**		**Lymphocyte absolute count 1** **×** **10**^****3****^**/μl**	
		**Median**	**IQR**	**Median**	**IQR**	**Median**	**IQR**
**H.D**.	40	1.77	1.37–2.16	3.61	2.85–4.14	1.90	1.60–2.33
**Active TB**	71	3.71	2.38–5.74	5.27	4.10–7.00	1.50	1.10–2.00
**Cured TB**	38	2.08	1.47–2.95	3.90	2.88–4.62	1.76	1.52–2.22

We then evaluated whether the increased N/L ratio found in patients with active TB disease was dependent on changes in the numbers of lymphocytes, neutrophils, or both. As shown in Table 2 and Figure 1B, N/L ratio was consistent with the associated significantly higher absolute neutrophils count (Median: 5.27, IQR: 4.1–7.0) but slightly (and significantly) lower absolute lymphocyte counts (Median: 1.5, IQR: 1.1–2), compared to H.D., and cured TB patients, reaching the higher statistical significance at *p* < 0.001 (Figure 1C). Moreover, there was a significant correlation between the N/L ratio and absolute neutrophil (*R*^2^: 0.53, *r*: 0.76, *p* < 0.0001) and lymphocyte (*R*^2^: 0.469, *r*: −0.72, *p* < 0.0001) counts, indicating that both the neutrophil and lymphocyte counts contribute to the altered N/L ratio (Figures 1D,E).

### Comparison of the Percentage and Phenotype of Circulating Neutrophils During Mycobacterial Infection

Detection of distinct circulating subsets displaying neutrophil-like morphology and showing immunosuppressive or proinflammatory functions has been well-documented in systemic inflammation, autoimmune diseases, and cancer (21–23). Here, we have evaluated the frequencies and phenotypes of two distinct subsets of neutrophils, NDNs and LDNs population isolated by density-gradient centrifugation of PBMCs from TB patients and H.D.

**Figure 2:**
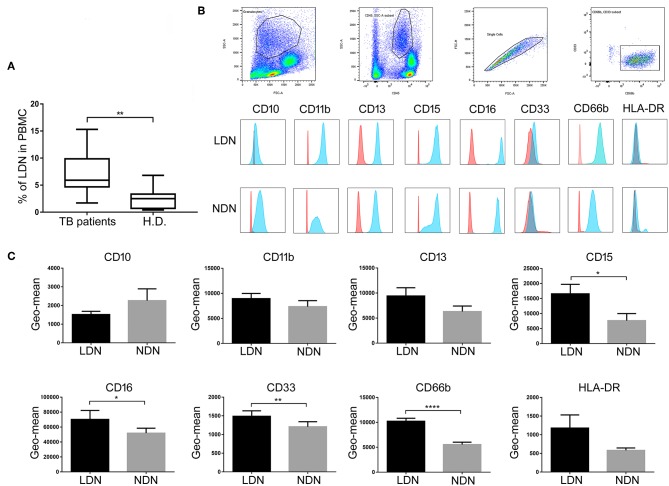
Frequency and surface molecules expression on neutrophil subsets population. **(A)** Box and whisker plots representing cumulative data of LDNs frequencies in active TB patients compared to H.D. Horizontal bars indicate the median, min, and max frequency of each group. Mann Whitney test was used to assess statistically significant differences between the groups, ^**^p < 0.01. **(B)** Representative FACS analysis of surface markers expression on circulating neutrophil subsets of one representative active TB patients. **(C)** Geometric mean fluorescence intensity (Geo-mean) of different surface molecules expressed on LDNs and NDNs of active TB patients (*n* = 22). Data are expressed as geo-mean ± SE. Student's *t-*test was used to assess statistically significant differences between NDNs and LDNs. ^*^p < 0.05, ^**^p < 0.01, ^****^p < 0.0001. Red histogram represents the Geo-mean of the isotype mAb control. Blue histogram represents the Geo-mean for each myeloid marker expression evaluated with each moAb used.

As shown in Figure 2A, PBMCs of patients with active TB disease contained significantly elevated percentages of LDNs compared with the PBMCs of control H.D. (5.9 ± 1.7% vs. 2.5 ± 0.40%, *p* < 0.01). Cell surface analysis showed that the LDNs in PBMCs of TB patients and H.D. stained as CD33^+^, CD66b^+^, CD11b^+^, CD10^+^, CD15^+^, CD13^+^, CD16^+^, and HLA-DR^+^, as reported in previous reports (24). Wright-Giemsa staining showed that the great majority of cells in this population (>95%) displayed a neutrophil morphology (Supplemental Figure 1). We then used these cell surface markers to evaluate LDNs in total blood and to compare the phenotypes of LDNs and NDNs by measuring the geometric mean fluorescence intensity (Geo-MFI). Figure 2B shows a representative analysis of surface markers expression in terms of Geo-MFI in one patient with active TB. As shown in Figure 2C, both neutrophil populations expressed the myeloid surface markers CD33, CD66b, CD11b, CD10, CD15, CD13, CD16, and HLA-DR, but LDNs showed significantly elevated expression of CD66b, CD33, CD15, and CD16 cell surface molecules, when compared to NDNs population.

### Functional Characteristics of LDNs and NDNs in TB Patients

**Figure 3:**
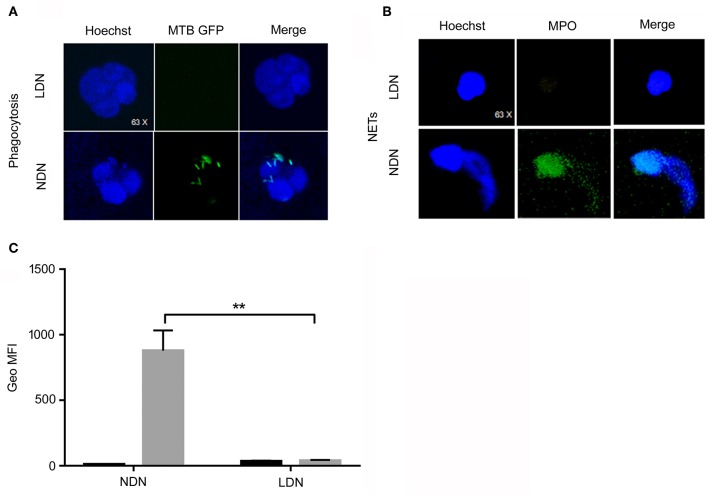
Functional analysis of LDNs and NDNs by confocal microscopy and flow cytometry. **(A)** LDNs and NDNs confocal microscopy of GFP-Mtb H37rv phagocytosis. Nuclei were stained with Hoechst (blue color) while GFP-*M. tuberculosis* were stained in green color. Merge of images shows no phagocytic activity by LDNs, while NDNs are able to phagocyte *M. tuberculosis*. **(B)** LDNs and NDNs confocal microscopy of NETs were analyzed for their ability to release NETs upon incubation with PMA. MPO was used as molecule that colocalize in conjunction with DNA *in vitro*. Nuclei were stained with Hoechst (blue color), while MPO molecules were stained in green color. **(C)** Flow cytometry representative data of oxidative burst test performed with the DHR123 method of NDNs (left side) and LDNs (right side) unstimulated or stimulated with PMA. The NDNs subset shows a normal oxidative burst activity while in the LDNs subset, PMA stimulation did not induce any oxidative burst. Bar graphs show cumulative data of Mean Fluorescence Intensity (MFI) of DHR123 in NDNs and LDNs stimulated with PMA (gray column) or unstimulated (black column). Multiple *t*-test was used to compare the difference among the two subsets; ^**^p < 0.01.

We first evaluated the capability of neutrophils to internalize *M. tuberculosis*. LDNs and NDNs were isolated from PBMCs as described in Materials and Methods and exposed for 3 h to *M. tuberculosis* H37Rv expressing green-fluorescent-protein (GFP). The “GFP positivity” of neutrophils was assessed by confocal microscopy and was used as a measurement of the phagocytic capacity of neutrophils. Highly suggestive of the lack of phagocytic capacity, LDNs failed to internalize GFP-tagged *M. tuberculosis*, while NDNs were fully capable to phagocyte the bacilli (Figure 3A). Moreover, we have evaluated the ability of the two different subsets of neutrophils to make NETosis upon *in vitro* stimulation with Phorbol 12-myristate 13-acetate (PMA), a potent activator of Protein Kinase C in Neutrophils. Figure 3B shows the ability of NDNs to make NETs, while the LDNs neutrophils do not display this capability.

To assess the bactericidal potential of LDNs and NDNs, we measured their ability to produce reactive oxygen species (ROS) in response to PMA stimulation, using the oxidation-sensitive fluorophore dihydrorhodamine-123s (24). As shown in Figure 3C, PMA treatment significantly enhanced the oxidative burst of NDNs, as compared to untreated cells (*p* < 0.01). Conversely, LDNs did not display any oxidative burst, as compared to the unstimulated cells. These results highlight that LDNs, differently from NDNs, lack both phagocytic and antimicrobial activities.

### Transcriptomic Profile of LDNs and NDNs From TB Patients

**Figure 4:**
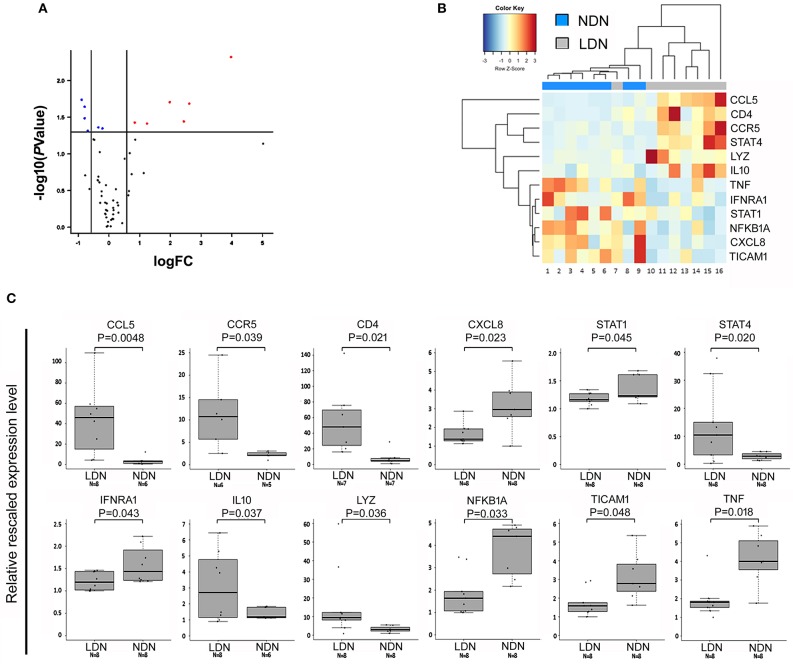
Genes differentially expressed in NDNs and LDNs of TB patients. **(A)** Volcano plot representing genes differentially expressed between LDN and NDN groups. Red and blue dots indicate significantly up- and down-regulated genes, respectively. FC, fold change. **(B)** Heat map representing the dysregulated genes between NDNs and LDNs of TB patients. Normalized median-centered expression values were used. Red and blue color intensities indicate up-regulation and down-regulation, respectively. The dendrogram clustering on the X-axis indicates sample similarity, whereas the dendrogram clustering on the Y-axis groups genes with similar expression profiles. Colored bars on the X-axis correspond to NDN and LDN groups. **(C)** Boxplots show expression levels of the 12 genes resulted significantly dysregulated in NDNs and LDNs of TB patients. Measurements were performed by semi-quantitative real-time RT-PCR assays (using the RT2 Profiler PCR Arrays). Boxes define the interquartile range; the thick line refers to the median. Results were normalized to expression levels of *ACTB* and *B2M* housekeeping genes and are presented as rescaled values. The number of subjects belonging to each group is indicated (*N*). Significance levels were calculated by the RT^2^ Profiler PCR Array Qiagen data analysis software.

NDNs and LDNs were isolated from 8 TB patients after gradient-density centrifugation of peripheral blood. The LDNs were further sorted by FACSAria as CD15^+^CD33^+^ population within the PBMCs. The expression of 84 cytokine and chemokine genes for each of the NDNs and LDNs cellular populations was analyzed and compared. Twelve genes were differentially expressed in the two neutrophil subsets: *CCL5, CCR5, CD4, IL10, LYZ*, and *STAT4* were significantly upregulated in LDNs when compared to NDNs (fold increase ranging from 1.8 to 15), while *CXCL8, IFNAR1, NFKB1A, STAT1, TICAM1*, and *TNF* were significantly downregulated in LDNs, as compared to NDNs (fold change ranging from 0.5 to 0.8; Figures 4A–C).

### LDNs Suppress or Do Not Have Any Effect on the Adaptive T Cell Response

**Figure 5:**
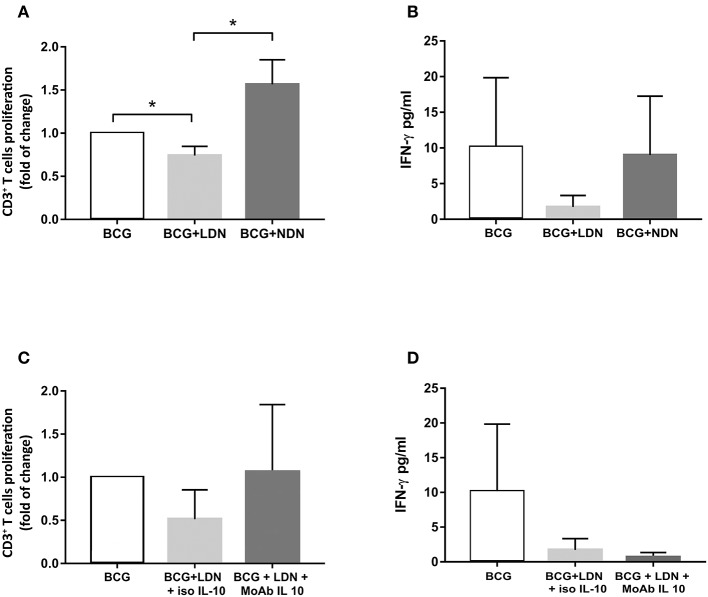
Inhibitory activity of LDNs and NDNs on T cell proliferation and IFN-γ production. **(A)** Bar graphs represent the cumulative data of proliferation, expressed as fold of change, of BCG-stimulated T cells (white column) in the presence of LDNs (gray column) or NDNs (dark gray column). T cell proliferation was evaluated on the CFSE negative CD3^+^ T cells fraction by FACS analysis and expressed as fold of change calculated as indicated in Materials and Methods section. **(B)** Bar graphs represent cumulative data of IFN-γ production, by BCG-stimulated PBMCs (white column) in the presence of LDNs (gray column) and NDNs subsets (dark gray column). **(C)** Bar graphs represent the cumulative data of proliferation of BCG-stimulated PBMCs (white column) in the presence of LDNs (gray column) and an isotype of control and LDNs (dark gray) in the presence of a neutralizing anti-IL-10 moAb. **(D)** Bar graphs represent the cumulative data, of inhibition of IFN-γ production of BCG-stimulated PBMCs (white column) in the presence of LDNs (gray column), in the presence of an isotype of control and LDNs (dark gray column), and in the presence of neutralizing anti-IL-10 moAb. Data shown are cumulative data from three different patients with active TB ^*^p < 0.05.

We evaluated whether circulating LDNs from TB patients displayed suppressive activities. To this aim, we assessed proliferation and interferon (IFN)-γ production of T cells from 3 TB patients in response to BCG stimulation, in the presence or absence of autologous LDNs or NDNs populations. As shown in Figure 5A, LDNs significantly inhibited T cell proliferation to BCG, when added to cultures at a ratio PBMCs/LDNs of 1:10, while NDNs had no inhibitory activity on T cell proliferation, as evaluated on the fraction of proliferating CD3^+^ T cells. Similarly, IFN-γ production by BCG-stimulated PBMCs from TB patients was not affected by addition of autologous NDNs, but was inhibited by addition of autologous LDNs, although differences did not reach statistical significance (Figure 5B). As transcriptomic analysis showed significantly upregulated *IL10* expression in LDNs, and given the well-known immunosuppressive effect of IL-10, we evaluated if the inhibition of T cell proliferation and IFN-γ production was mediated by IL-10. As shown in Figure 5C, addition to co-cultures of a neutralizing anti-IL-10 monoclonal antibody (moAb) partially restored the proliferation, but failed to restore IFN-γ production that were inhibited by LDNs (Figure 5D), indicating that the inhibitory activity of LDNs is not attributable to IL-10.

## Discussion

Neutrophils contribute to the immune response to *M. tuberculosis* infection and their numbers and functions positively correlate with the active disease stage in humans. Several studies have highlighted the contribution of neutrophils to lung pathology rather than a protective role (25). Moreover, in highly susceptible *M. tuberculosis* mouse strains, the pharmacological interference with neutrophil-associated inflammation or their depletion resulted in reduced pathology, increased survival rates and, ultimately, less mycobacterial burden (25). Several evidences identified neutrophils as potential targets for host-directed therapies as adjunct anti-tuberculosis treatment (26–28).

In the present study, we showed that patients with active TB disease had a very high N/L ratio, as compared to both H.D. as well as cured TB patients, suggesting that the N/L ratio is changed after anti-TB therapy and could be used as a tool to evaluate treatment success. In active TB patients, the N/L ratio was significantly correlated with increased neutrophils count and lower lymphocyte count, indicating that both populations contribute to the altered N/L ratio. Thus, our results agree with previous studies showing that the N/L ratio predicts efficacy of pulmonary TB treatment (11), but diverge from another study suggesting that N/L ratio may distinguish TB patients from tuberculin skin test-positive healthy contacts (29).

Like macrophages, neutrophils can be separated into different classes on the basis of functional characteristics. NDNs are typically considered phagocytic cells, whereas LDNs are considered phagocytosis-defective cells (17). However, the phenotype and physiological function of these populations remain elusive. Therefore, we became interested in assessing NDNs and LDNs in patients with active TB disease. We found that the amount of LDNs in the blood of TB patients was significantly increased compared with that in the blood of H.D. Moreover, the expanded LDNs population expressed the same myeloid markers as the NDNs population, with the expression of CD16 and CD11b delineating a mature cellular neutrophil population. The results presented here are consistent both with the study of Vollbrecht et al. demonstrating an expansion of CD15^+^CD33^+^CD11b^+^ population in HIV-1 infection (30), and with the study by Cloke et al. describing a population of activated LDNs in PBMCs of HIV-1-infected patients (17).

However, in contrast with several previous studies in cancer, but consistently with a recent study by de Kleijn and colleagues (31), we demonstrate that the LDNs subset in the circulation of TB patients express significantly elevated levels of CD66b, CD33, CD16, and CD15, as compared to the NDNs subset.

Several studies have suggested that not only the phenotype, but also the functions of LDNs may vary depending on the disease context. The expanded LDN population in TB patients did not produce ROS upon stimulation with PMA and failed to internalize *M. tuberculosis*, in sharp contrast to the NDNs subset with either produced ROS and internalized *M. tuberculosis*. Many pathogens, namely viruses, bacteria, parasites, and fungi can induce NETs. For this reason, the mechanisms for both the initiation and evasion of NETs by pathogens have been intensively studied (32). Moreover, NETosis induction depends on the generation of ROS by myeloperoxidase (33). A previous study described that *M. tuberculosis* infection triggered NETosis. Data here reported demonstrate that LDNs are not able to induce NETosis, in contrast to the NDNs subset.

Therefore, the expanded LDNs population during active TB seems to possess unique phenotypic and functional features. This was further strengthened by transcriptomic analysis of LDNs and NDNs purified from the peripheral blood of TB patients, that identified 12 differentially expressed genes. From this analysis we found that the upregulation of *IL10* could be related to a regulatory role of the LDNs population during active TB. In fact, it is known that neutrophils can modulate the functions of a variety of T cell subsets, including subpopulations of CD4^+^ αβ T cells [Th1 cells, Th17 cells, Th2 cells, and T regulatory (Treg) cells], CD8^+^ αβ T cells and γδ T cells, either *in vitro* or *in vivo*, in both humans and mice (33–37). The immunosuppressive role of certain subsets of neutrophils, such as LDNs and/or myeloid derived suppressor cells (MDSCs), has gained most of the attention in several contexts (38, 39). In our study, LDNs displayed immunosuppressive activities toward antigen (BCG) activated T cells, inhibiting both their proliferative and IFN-γ responses. Our findings are in agreement with two recent studies (39, 40), which showed that the most suppressive subset of human LDNs/MDSCs belong to the mature neutrophil population, at least in healthy volunteers receiving G-CSF for stem cell mobilization for bone marrow transplantation (41), or in cancer patients, respectively (42).

On the other hand, the regulatory function of neutrophils was identified in several models. Neutrophils isolated from both bronchoalveolar lavage fluid and parenchyma of *M. tuberculosis* infected mice produced IL-10 which downregulated local lung inflammation during chronic phase of infection (35). In contrast, the down-modulation of IFNAR1 could be related to the low neutrophil activation in terms of cytokines and chemokines release that contribute to the inflammatory response. Moreover, the downregulation of the transcription factors NFKB1A, STAT1, and TICAM1 could be related to the less inflammatory/protective role during active TB disease.

In our study, however, IL-10 does not participate to the inhibitory activities of LDNs, as indicated by the failure of a neutralizing anti-IL-10 moAb to rescue proliferative and IFN-γ responses in co-cultures of T cells and LDNs suggesting that other mediators are involved. Thus, further investigation is required to define the mechanisms of immunosuppression of T cell responses by LDNs during TB.

In summary, biological properties of the two isolated neutrophil populations suggest their dual role during TB: NDNs provide a mechanism for bacteria killing, through oxidative burst and NETosis, by upregulating transcription factors involved in the release of cytokines and activation of innate and acquired immune cells, LDNs instead exert suppressive activities on T cell response. The balance between these two subsets of neutrophils might influence either the initial steps of innate immune responses or the subsequent development of the adaptive immune response to *M. tuberculosis*, ultimately influencing the outcome of infection.

## Data Availability Statement

The genetic data generated for this study were deposited in the repository Geobank access code GSE138948 (https://www.ncbi.nlm.nih.gov/geo/query/acc.cgi?acc=GSE138948), and the other datasets generated for this study are available on request to the corresponding author.

## Ethics Statement

The study was approved by the Ethical Committee of the University Hospital in Palermo (Approval No. 13/2013), where the patients were recruited. The patients/participants provided their written informed consent to participate in this study.

## Author Contributions

FD and NC conceived and designed the experiments and wrote the paper. ML, VO, BT, and EP performed the experiments. FD, NC, RA, EP, AC, and PD made intellectual contributions to the work. FD, NC, RA, EP, ML, and VO analyzed the data. PD, AC, BT, ML, and VO enrolled the patients and collected the clinical information. PD, BT, AC, and RA supervised the laboratory collection of the clinical samples. All authors have reviewed the manuscript.

### Conflict of Interest

The authors declare that the research was conducted in the absence of any commercial or financial relationships that could be construed as a potential conflict of interest.

## Supplementary Material

Supplemental Figure 1 Giemsa staining of LDN and NDNs neutrophils. NDNs and LDNs were stained with Giemsa: optical microscopy of **(A)** LDN and **(B)** NDN shows the different morphology of the nuclei.
