# Supplementary figures and images for: Mycobacterium tuberculosis Drives Expansion of Low-Density Neutrophils Equipped With Regulatory Activities
Source: Front Immunol. 2019 Nov 27;10:2761. doi: 10.3389/fimmu.2019.02761 (PMC6892966; doi:10.3389/fimmu.2019.02761)

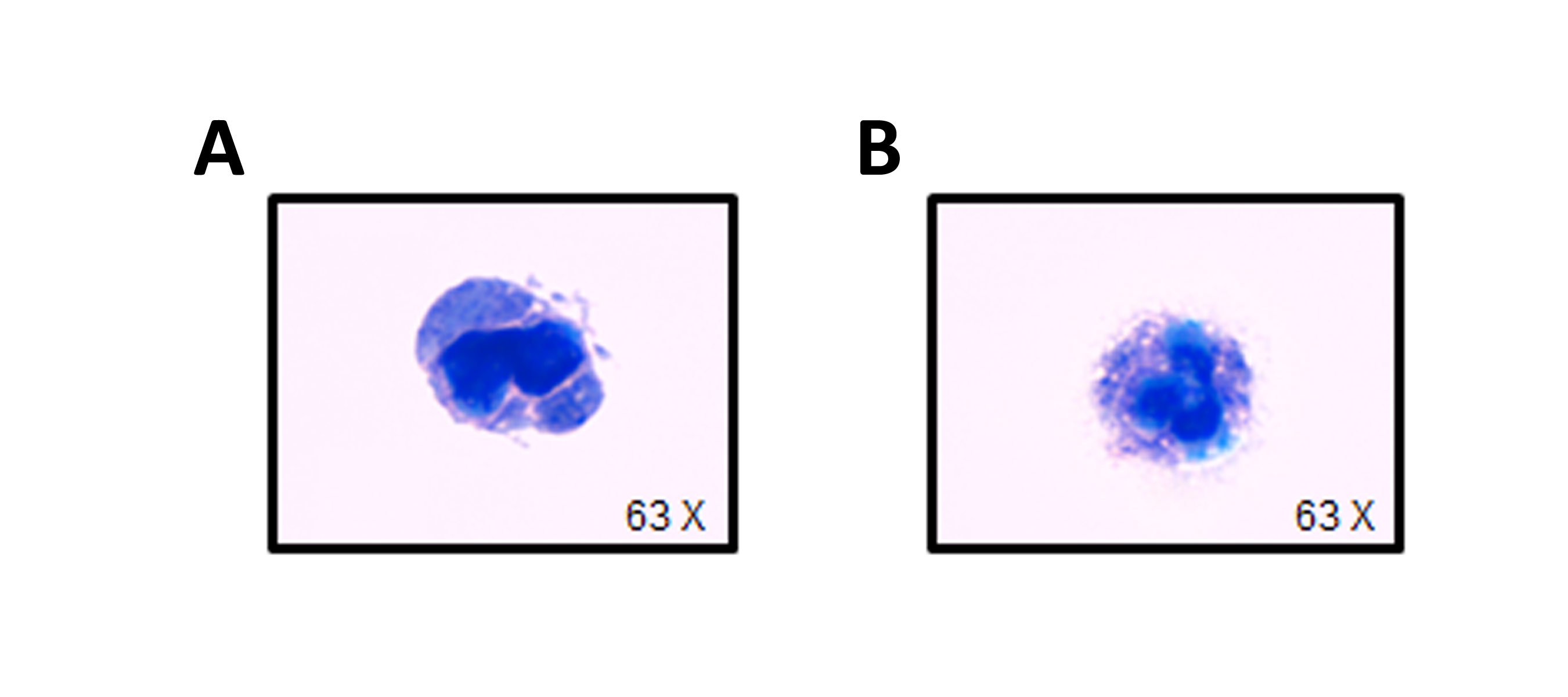

Supplement: Supplemental Figure 1 — Giemsa staining of LDN and NDNs neutrophils. NDNs and LDNs were stained with Giemsa: optical microscopy of (A) LDN and (B) NDN shows the different morphology of the nuclei. [file Image_1.TIF]
